# Supplementary figures and images for: Prognostic analysis of histopathological images using pre-trained convolutional neural networks: application to hepatocellular carcinoma
Source: PeerJ. 2020 Mar 12;8:e8668. doi: 10.7717/peerj.8668 (PMC7073245; doi:10.7717/peerj.8668)

A

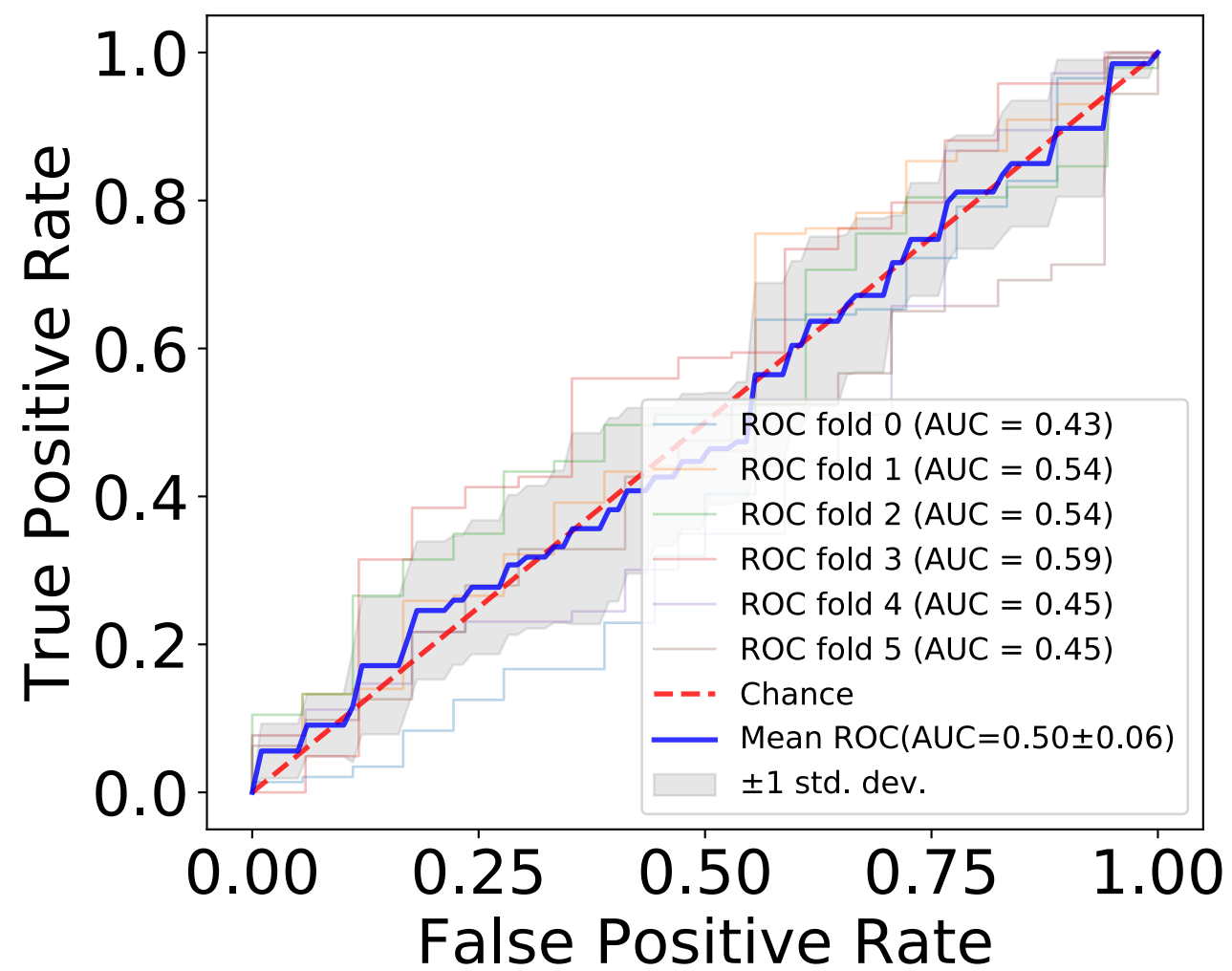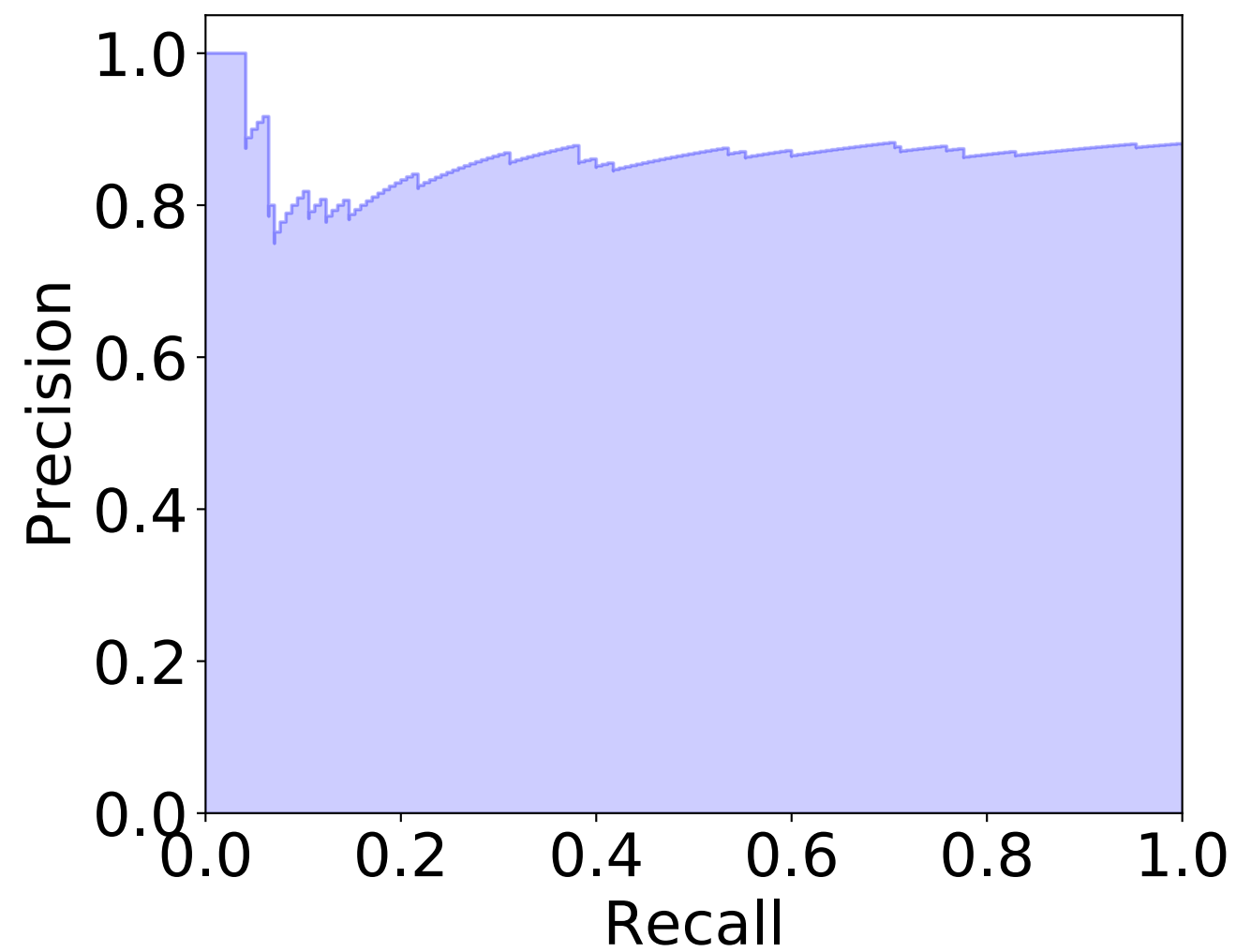

B

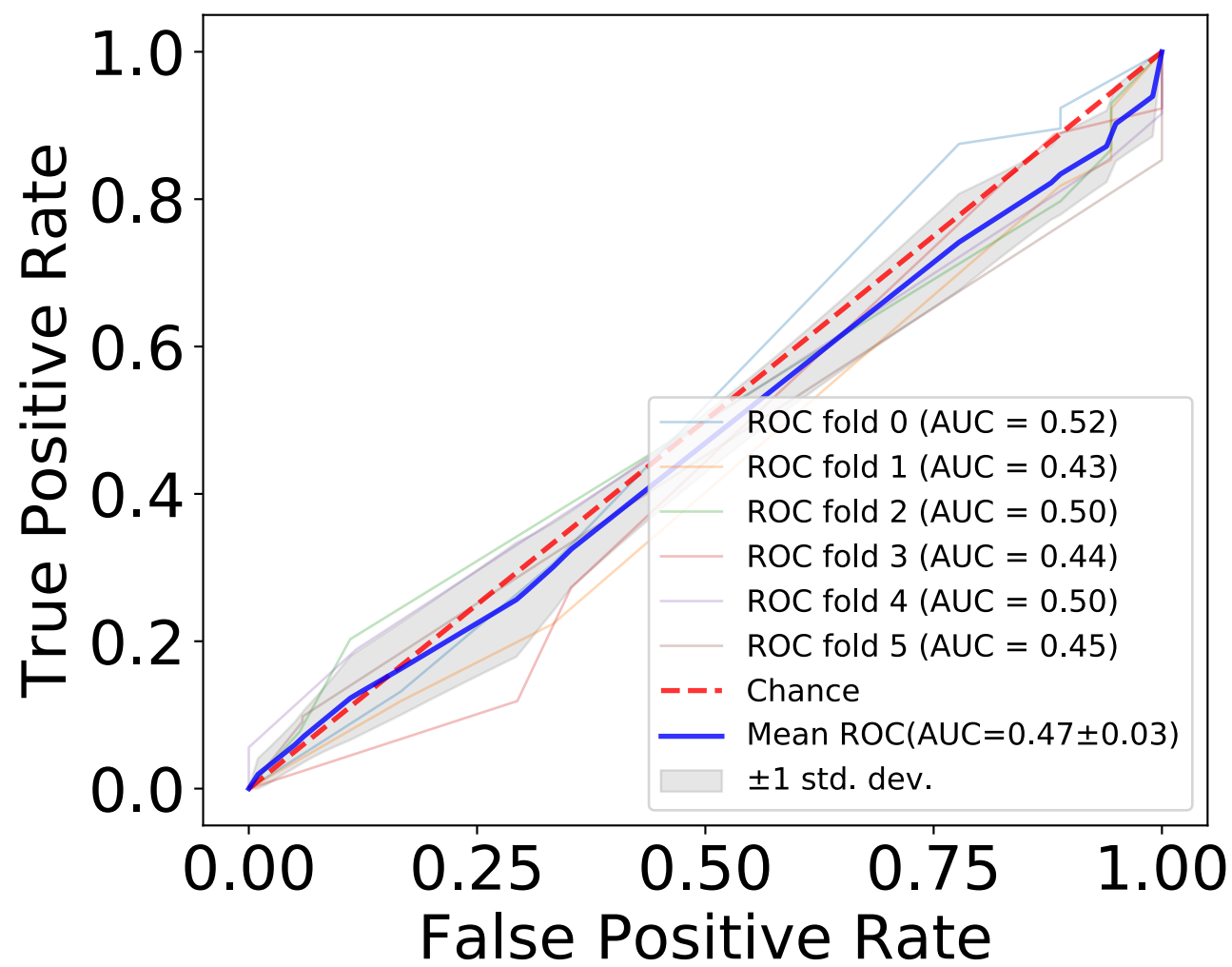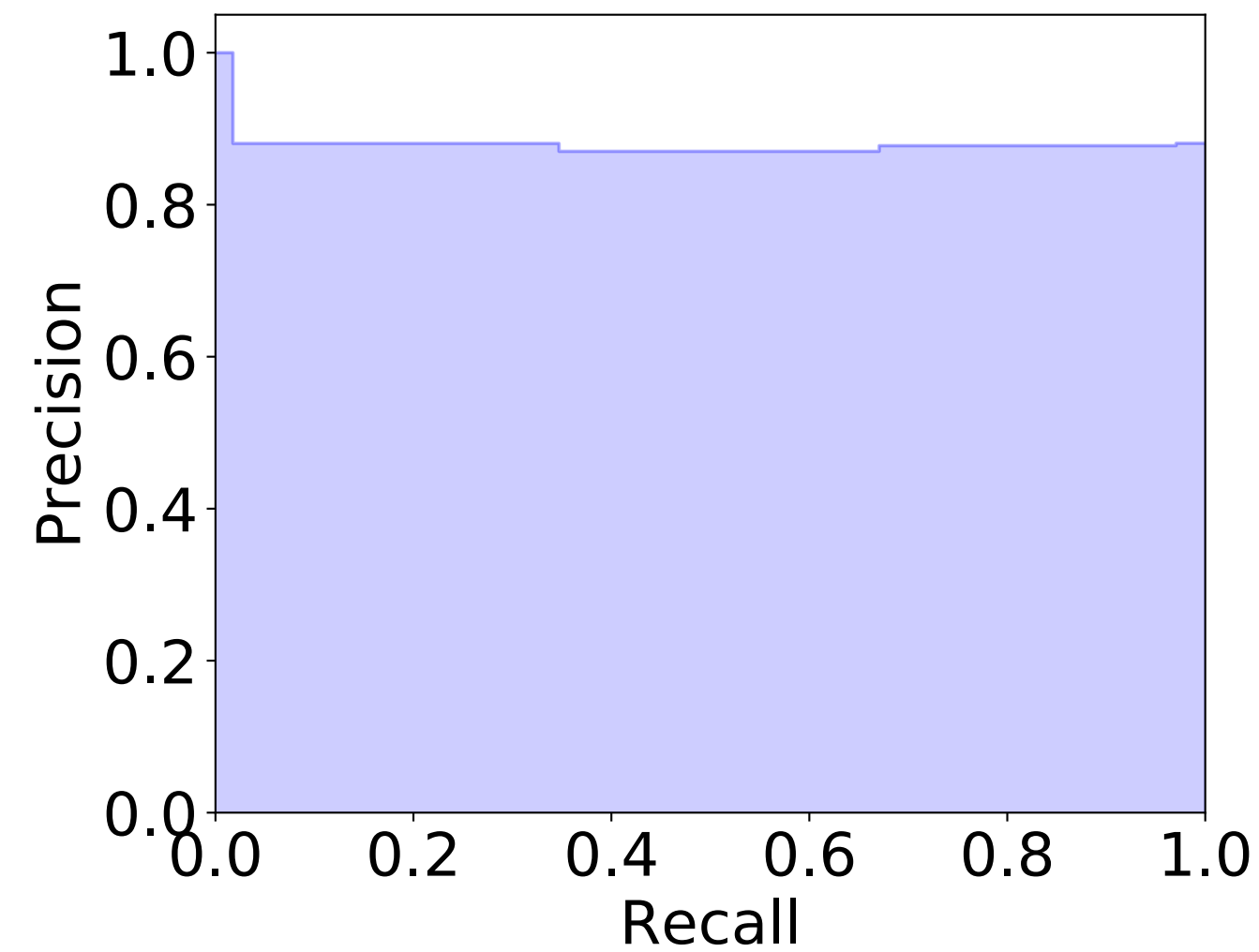

Supplement: Supplemental Information 1 — The left and right sides respectively show ROC curves and Precision-Recall curves from linear SVM classification. (A) PCA performance, (B) SVD performance. [file peerj-08-8668-s001.pdf]

**A**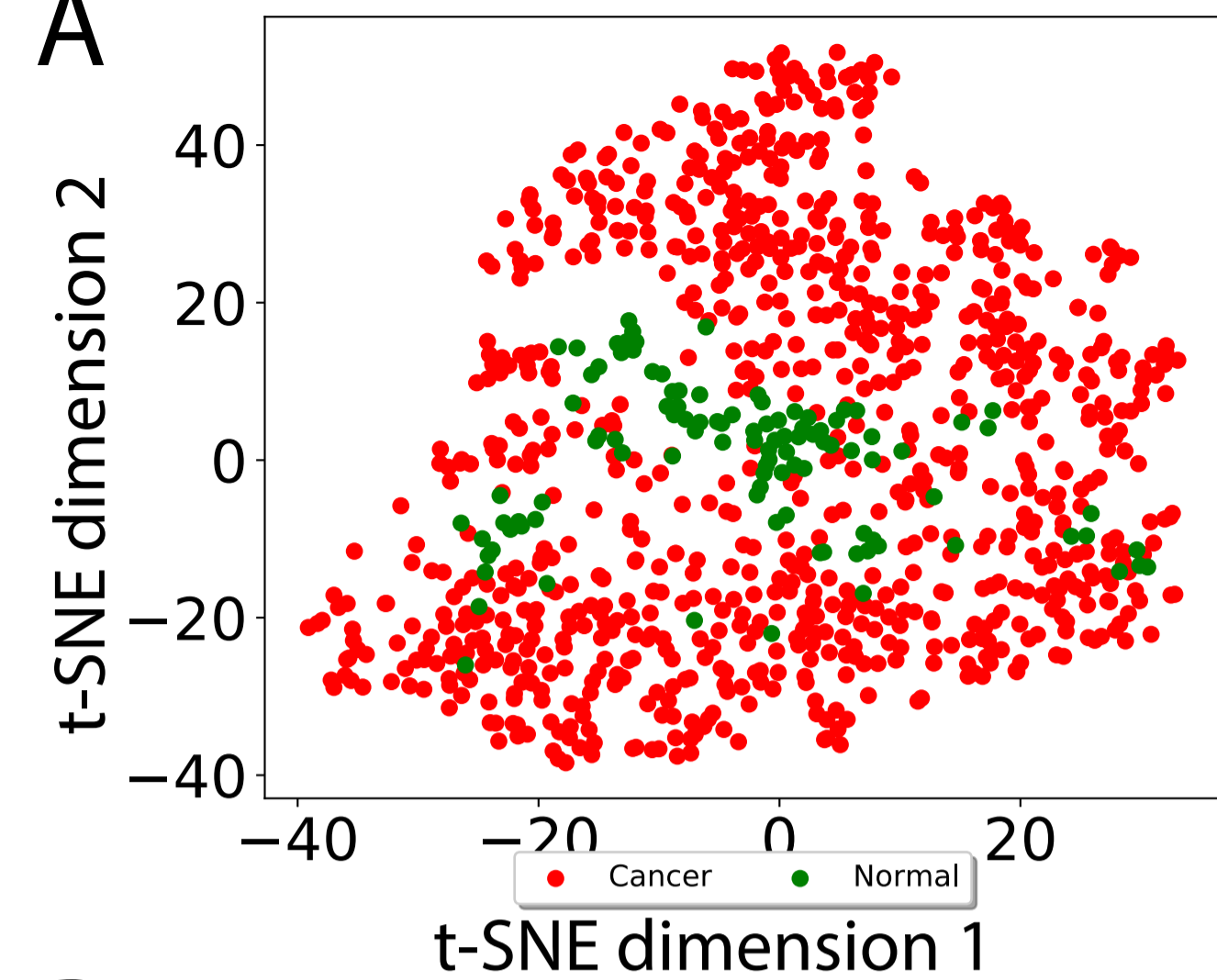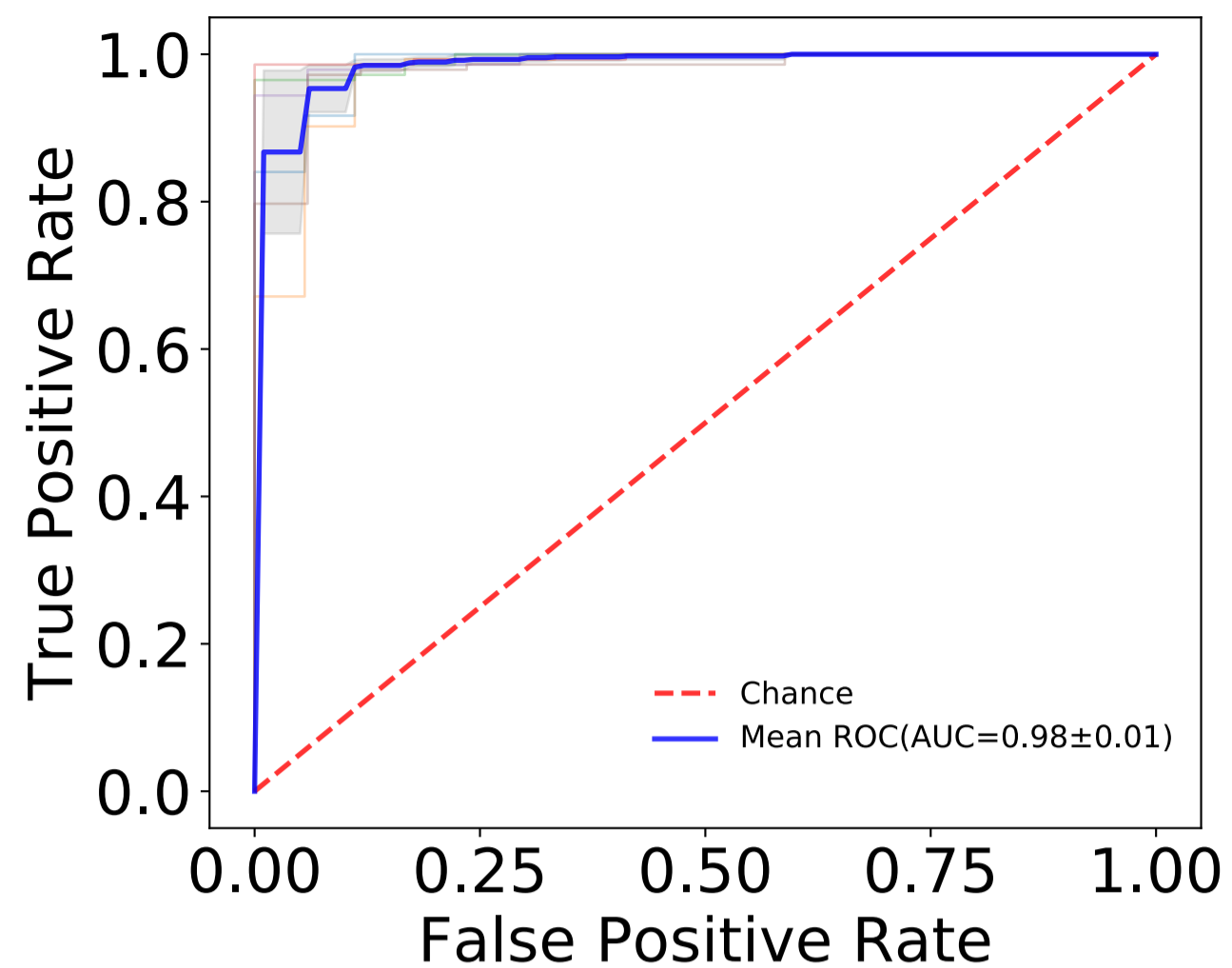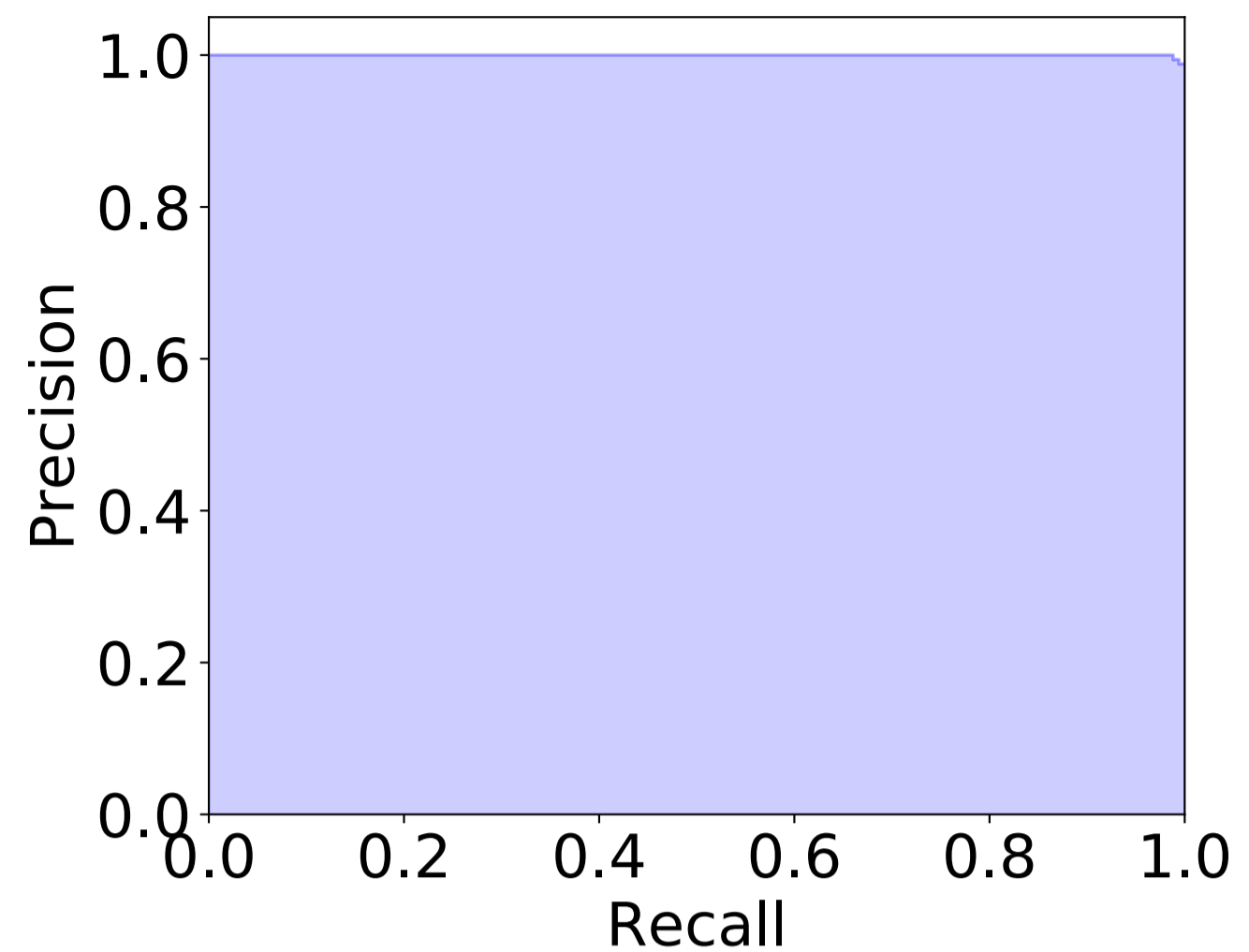**B**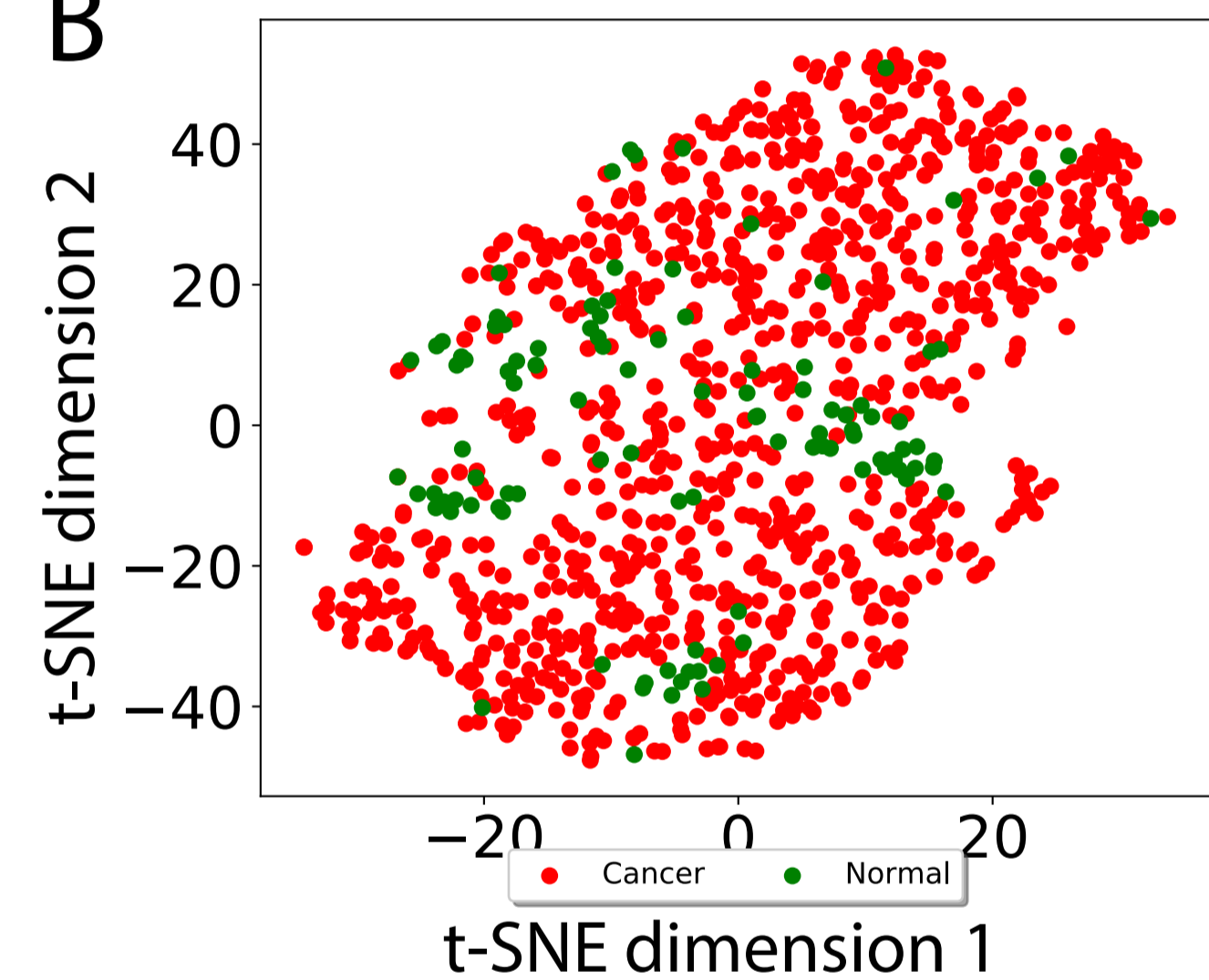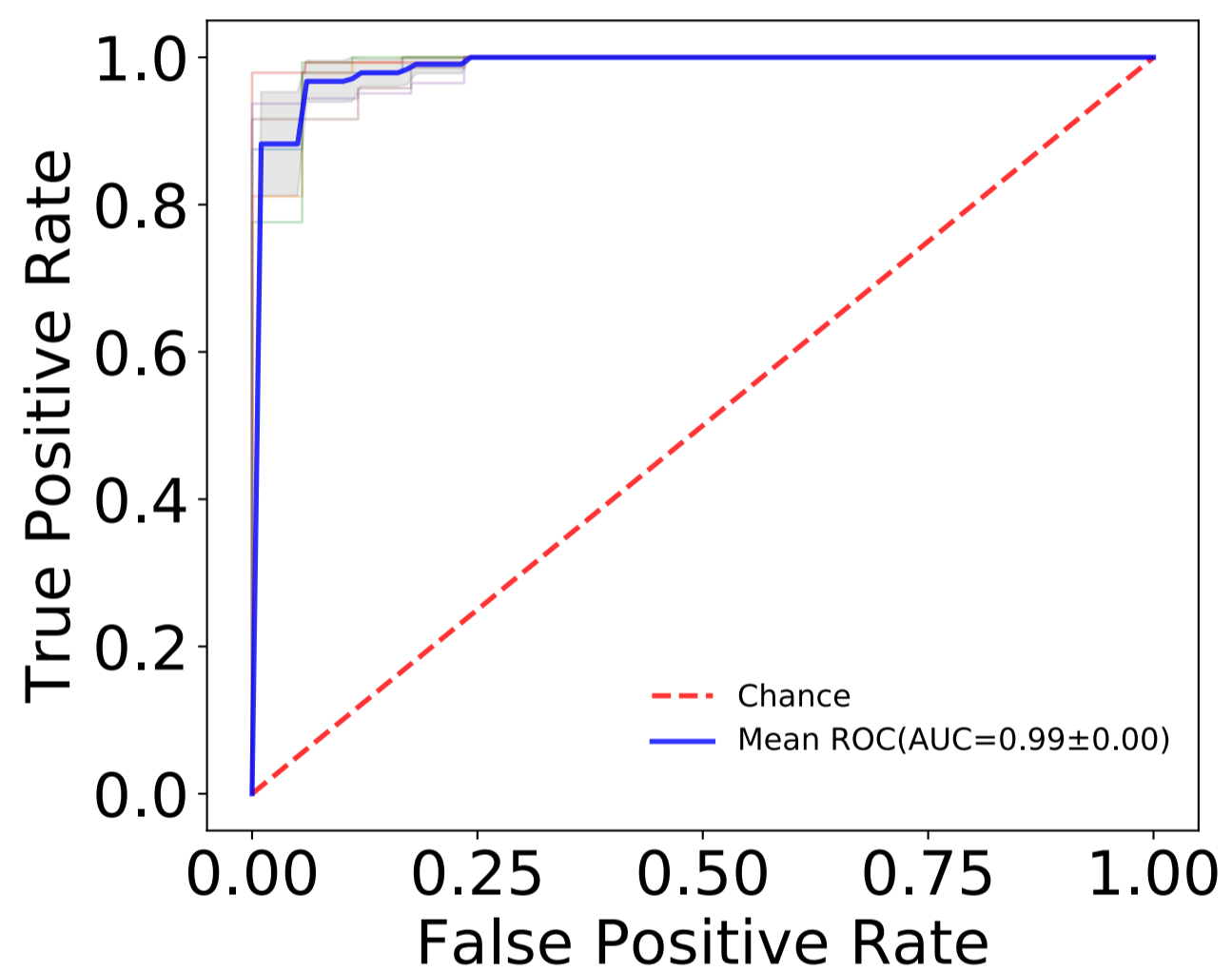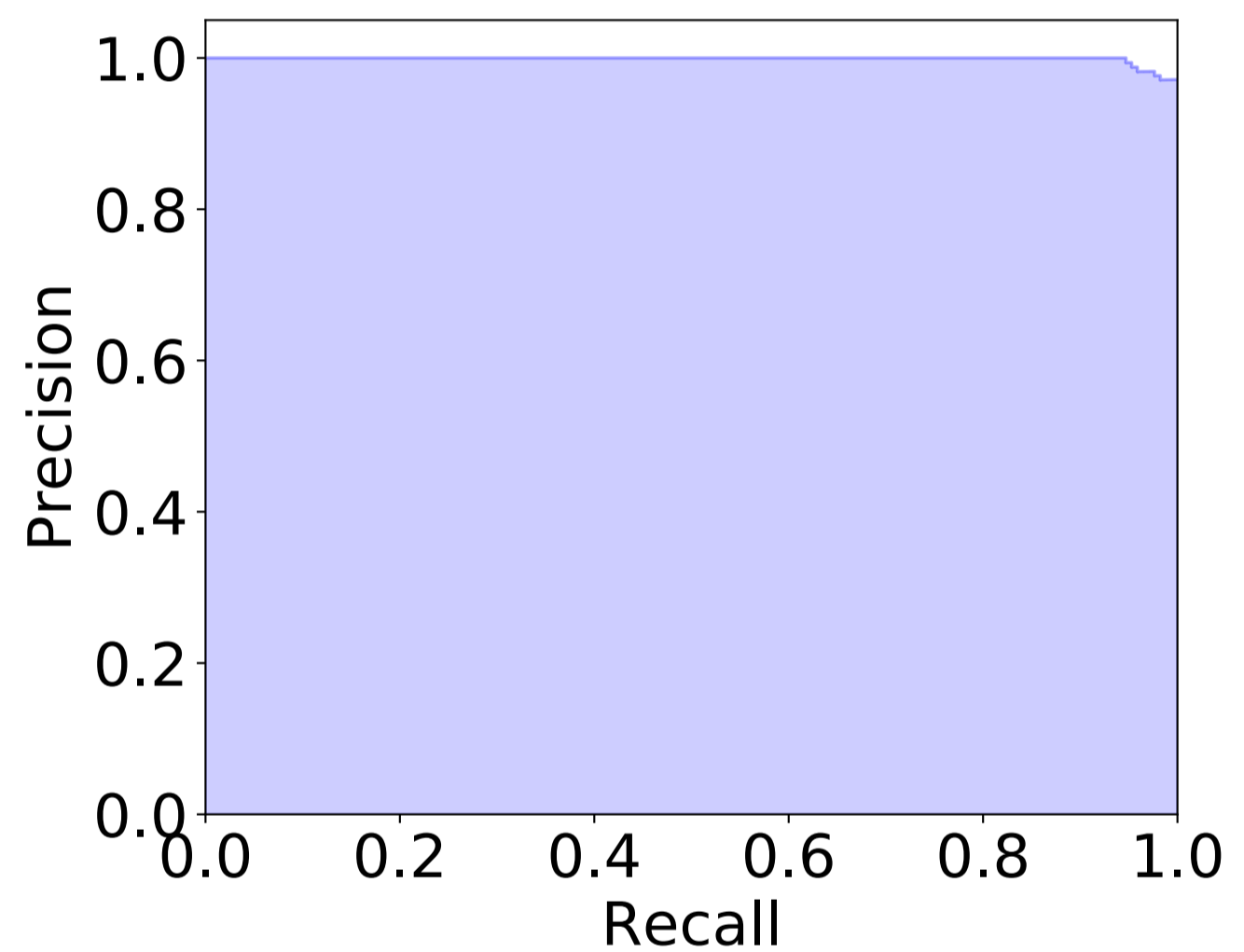**C**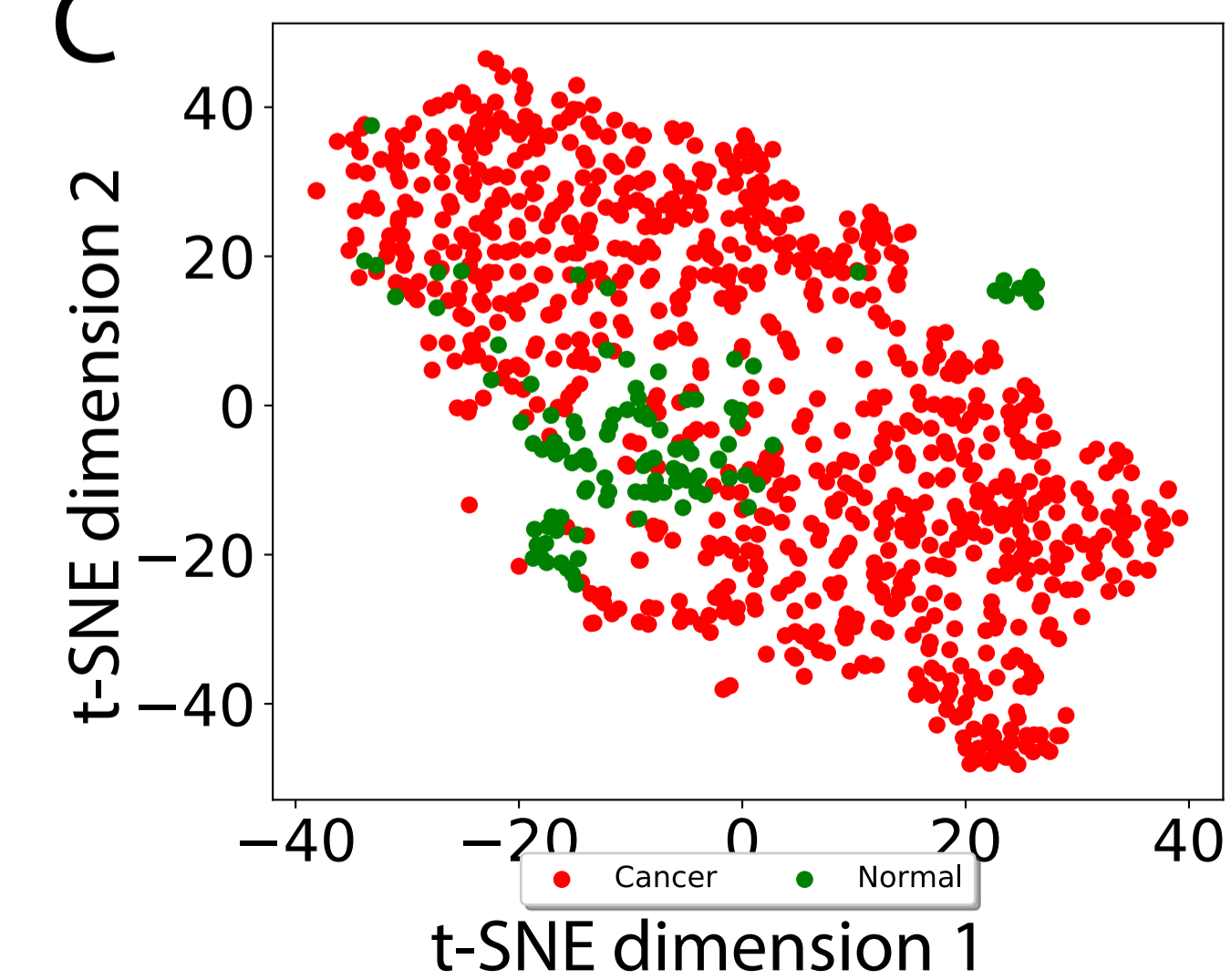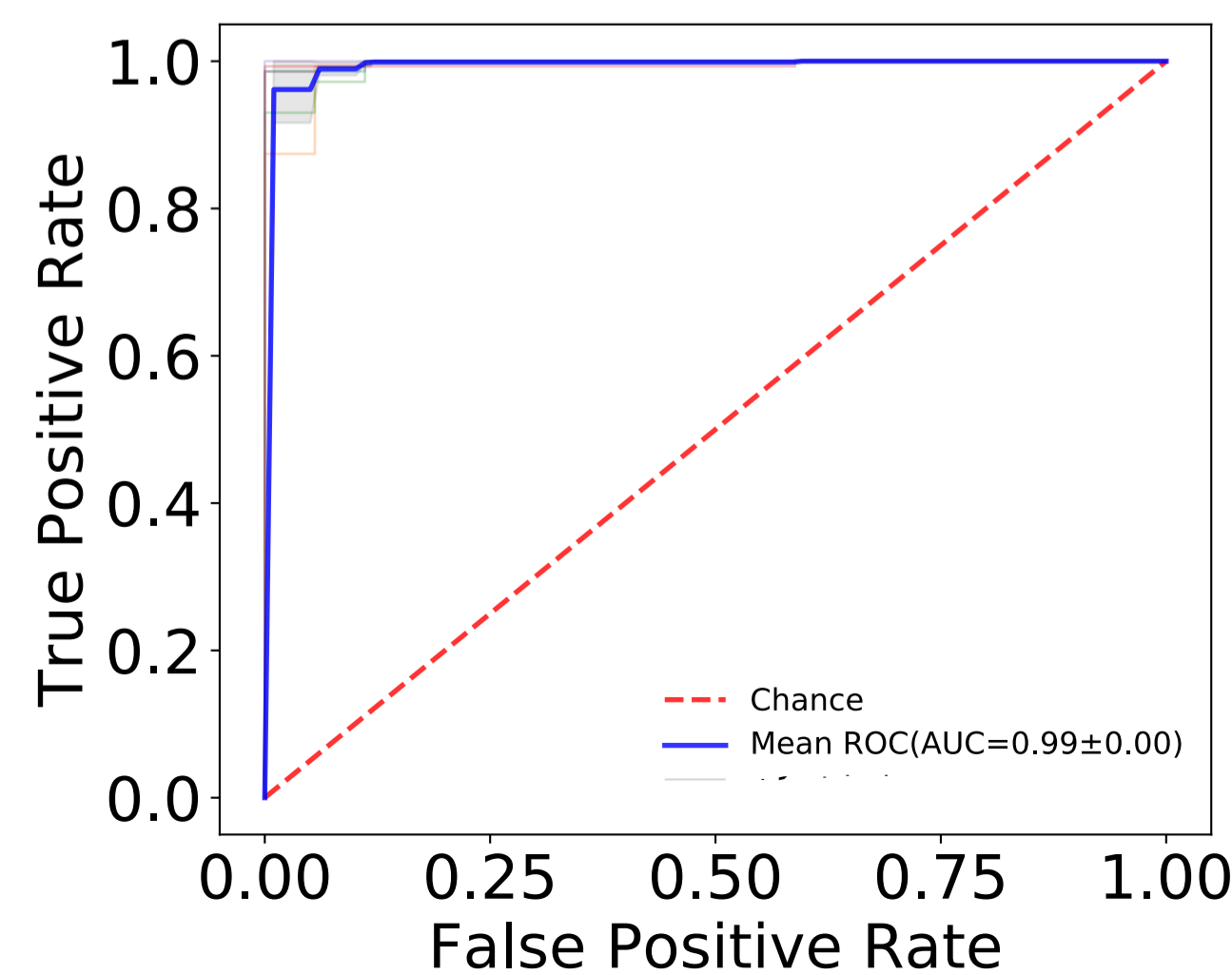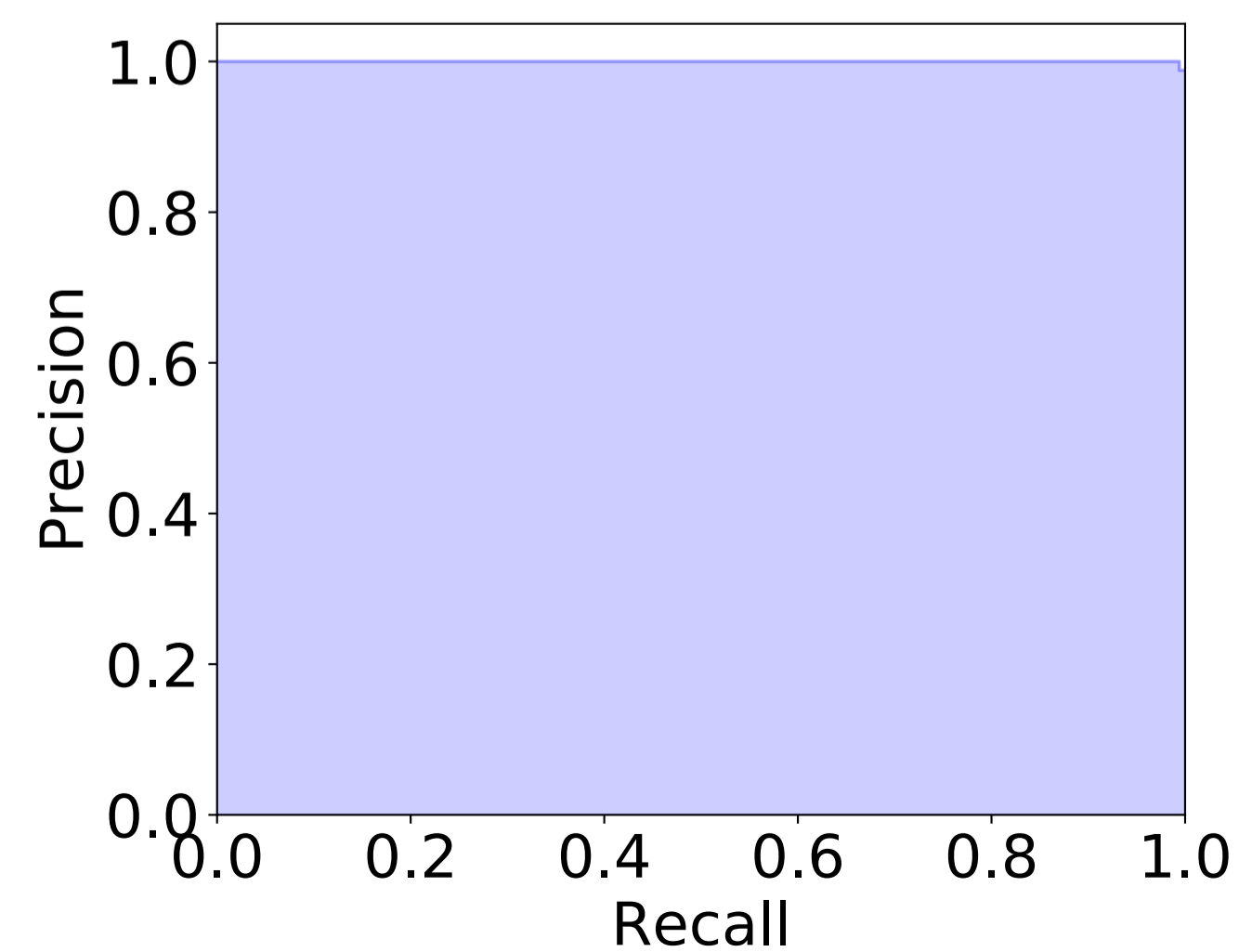

Supplement: Supplemental Information 2 — The left side indicates t-SNE visualization, the middle indicates ROC curves from linear SVM and the right side indicates Precision-Recall curves measured using (A) VGG image features, (B) Inception features, (C) ResNet features. [file peerj-08-8668-s002.pdf]

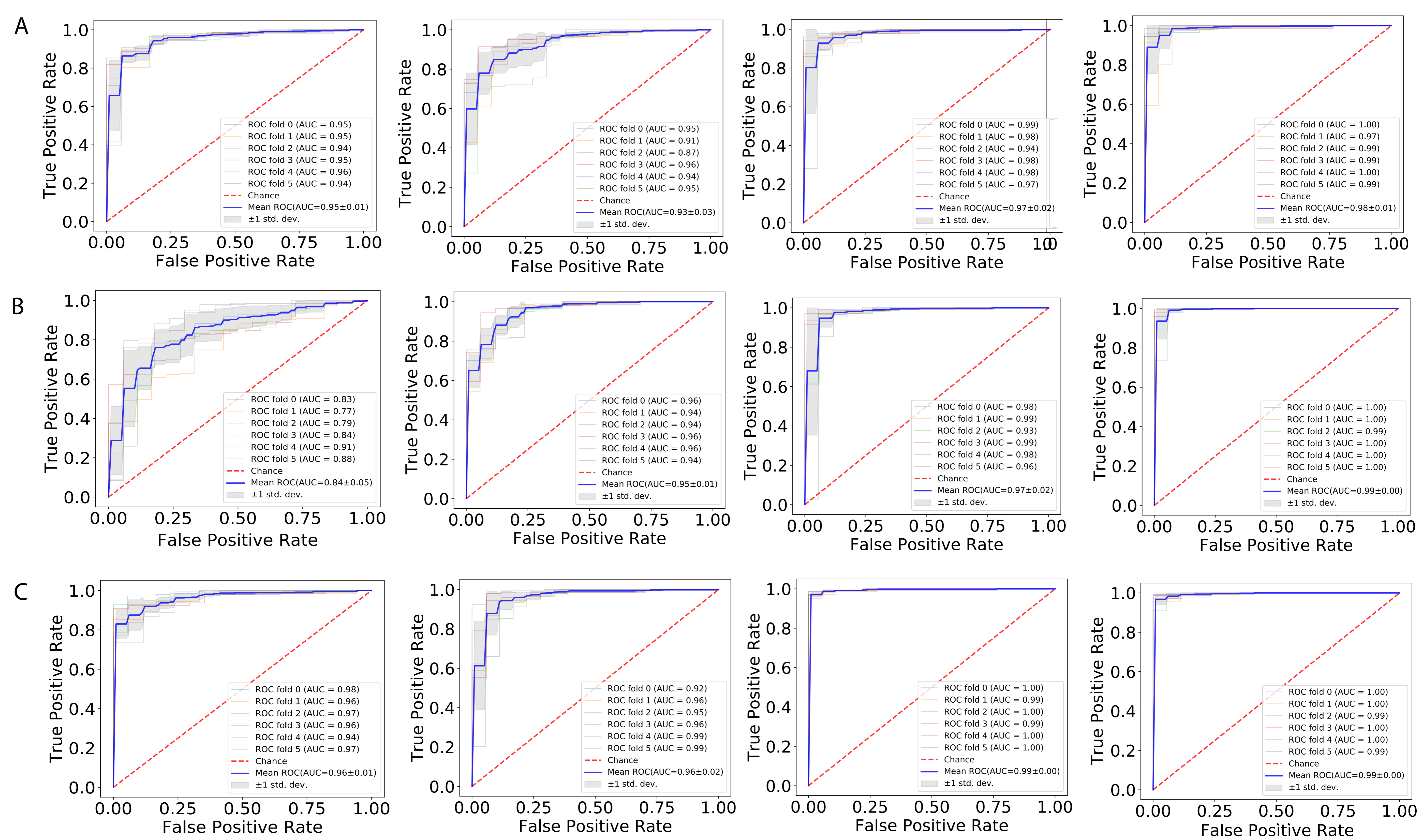

Supplement: Supplemental Information 3 — Classification ROC curves using crop size 256 × 256 from a series of randomly selected features of size 10, 25, 50 and 100 (left to right) from three models (A) VGG, (B) Inception and (C) ResNet. [file peerj-08-8668-s003.pdf]

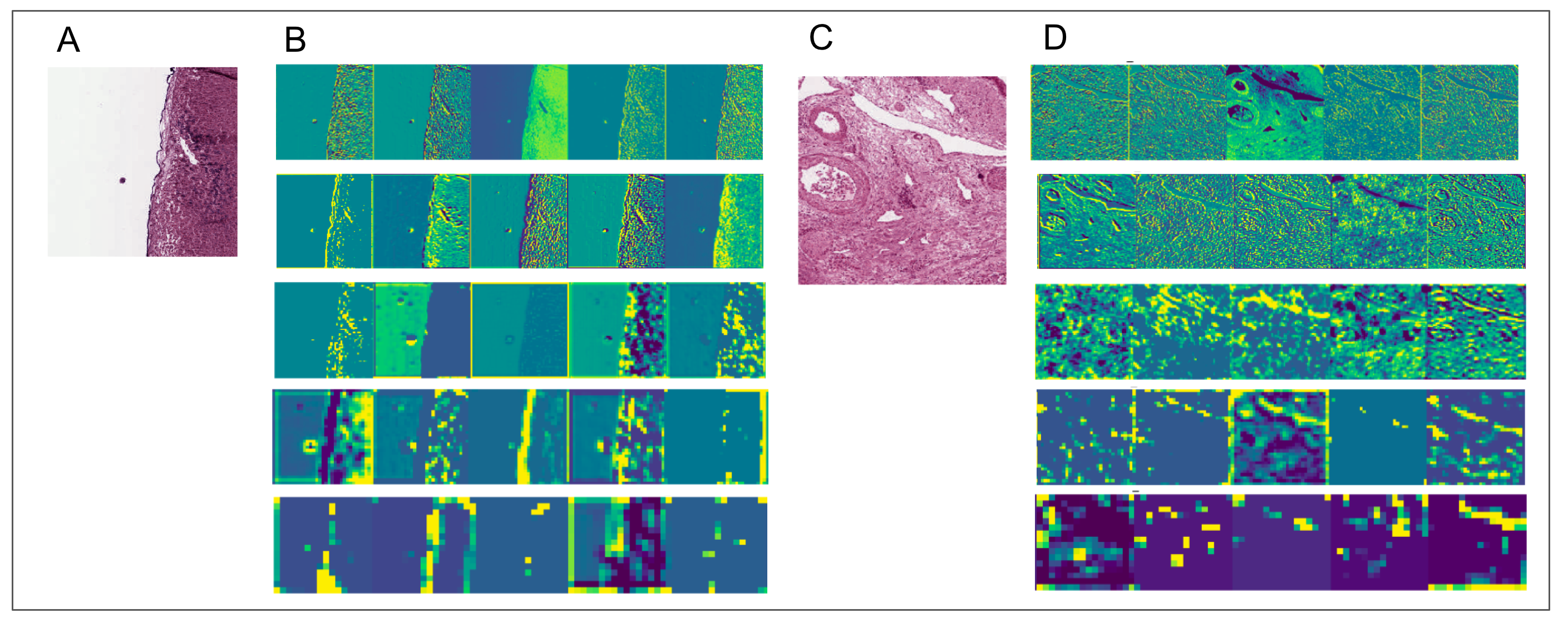

Supplement: Supplemental Information 4 — (A) and (C) show original images from a 256 × 256 pixel crop size, (B) and (D) visualize the feature mapping from convolutional block 1 (top row) to convolutional block 5 (bottom row) for the VGG16 model. [file peerj-08-8668-s004.png]

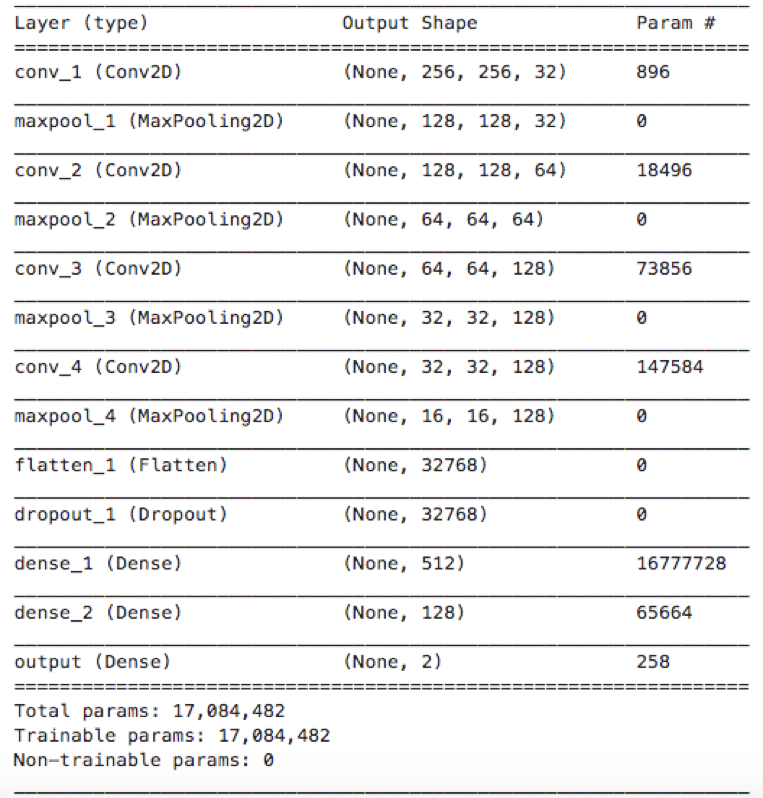

Supplement: Supplemental Information 5 — The model includes three convolutional and pooling layers, followed by two fully-connected layers. Two dropout layers were added in the fully-connected layers to avoid overfitting. The ADAM optimizer was applied for model training. Evaluation metrics for the model include binary cross entropy and accuracy. [file peerj-08-8668-s005.png]

Last Epoch - Train Loss: 0.185, Val Loss: 0.317

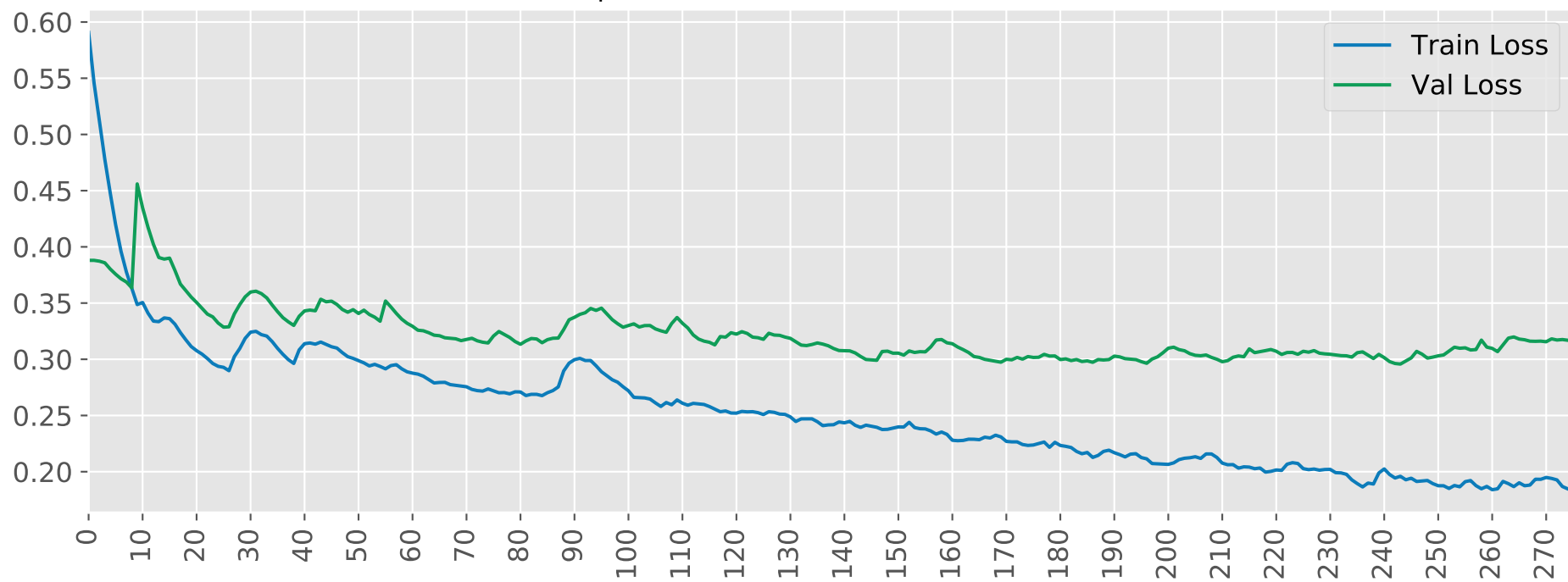

Last Epoch - Train Accuracy: 0.930, Val Accuracy: 0.862

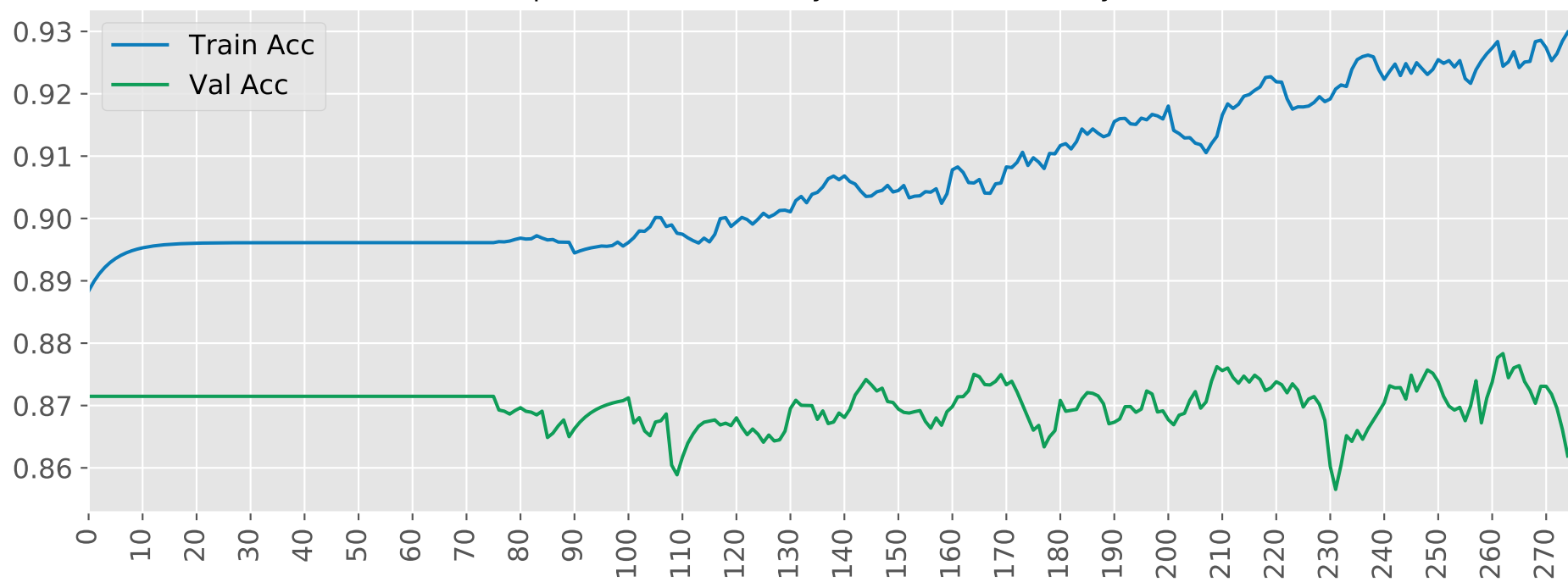

Supplement: Supplemental Information 6 — Within 275 epochs’ training, the accuracy for the last epoch was 0.930 and 0.862 for training and validation respectively. The best validation accuracy was 87.8%. [file peerj-08-8668-s006.pdf]
